# Treatment at the front end of the criminal justice continuum: the association between arrest and admission into specialty substance abuse treatment

**DOI:** 10.1186/1747-597X-1-20

**Published:** 2006-07-31

**Authors:** Sheryl Pimlott Kubiak, Cynthia L Arfken, James A Swartz, Alison L Koch

**Affiliations:** 1 School of Social Work, Wayne State University, Detroit, MI, USA; 2 Department of Psychiatry and Behavioral Neurosciences, Wayne State University, Detroit, MI, USA; 3 Jane Addams College of Social Work, University of Illinois at Chicago, Chicago, IL, USA

## Abstract

**Background:**

To reduce criminal recidivism and drug use, it has been proposed that the substance abuse treatment delivery system cut across different components of the criminal justice continuum. Arrest, at the front end of this continuum, may represent a critical moment to motivate people with substance use disorders (SUD) to seek treatment but is often over looked as an intervention point. We used data from the 2002 National Survey on Drug Use and Health (NSDUH) to compare treatment need and recent treatment admission for participants with no criminal justice (CJ) involvement in the past year, past-year arrest, and CJ supervision (i.e., probation or parole status).

**Results:**

Of those arrested, 44.8% met criteria for an SUD. However, only 14% of those arrested with an SUD received treatment in the year of their arrest. In multivariate modelling, arrest was an independent predictor of treatment admission (odds ratio (OR) = 8.74) similar in magnitude to meeting criteria for an SUD (OR = 8.22). Those further along the continuum – under supervision – were most likely to receive treatment (OR = 22.62).

**Conclusion:**

Arrest involves the largest number of individuals entering the criminal justice system. The NSDUH suggests that nearly 6 million individuals in the US experience an arrest annually and that nearly half meet criteria for an SUD. Although arrest involves the largest number of individuals entering the criminal justice system, it is also the most fleeting point as individuals can move in and out rather quickly. Minimally, arrest imposes contact between the individual and a law enforcement person and can be an opportunity for early intervention strategies such as pre-arraignment diversion into treatment or brief intervention strategies. Using brief intervention at this early point in the continuum may motivate a greater number of individuals to seek treatment or decrease drug and alcohol use. Training and procedural shifts at this point of contact could have important policy implications in reducing the number of subsequent arrests or preventing individuals moving further along the criminal justice continuum, as well as decreasing the fiscal and resource burdens associated with criminal justice processing and confinement.

## Background

Treatment of alcohol and drug disorders can be a critical step in reducing criminal recidivism [1-3]. However, even though data suggest that approximately 40% of men and women arrested meet criteria for drug dependency – highlighting the need for treatment services – few arrestees receive treatment in the year prior to their arrest [4].

In general, efforts to increase treatment seeking for alcohol or drug abuse treatment have focused on improving access as well as the use of brief interventions during critical moments such as medical crisis [5]. Arrest, like medical crisis, may be a catalyst for treatment seeking by motivating an individual contemplating change to actually take steps to initiate that change [6-8]. Similarly, arrest may mobilize other supports (e.g., a spouse, employer) to pressure the individual to enter treatment [9,10]. Alternately, an individual may enter treatment as a way to avoid or reduce negative criminal justice outcomes. For instance, an individual may pursue treatment prior to trial as a way to ingratiate oneself with the court. Moreover, even if charges are dropped or the individual is not convicted, arrest may be a catalyst for treatment as a way to avoid subsequent arrest.

When considered from the criminal justice perspective, there is a continuum of association with an individual that begins with arrest. Individuals may leave the continuum through charges being dropped or move along to arraignment, trial, and if convicted, sentencing. Sentencing options vary based on offense type, judicial discretion and/or legal statutes but generally range from community-based sanctions under criminal justice supervision (i.e. probation) to incarceration. Substance abuse interventions such as pre-trial diversion programs show promise, as strategies at earlier stages in the continuum [11-14], while prison-based treatment, such as therapeutic communities with aftercare, are successful strategies later in the continuum [15,16]. Each strategy attempts to reduce drug abuse and further involvement in the criminal justice system. Many communities have interventions at one or more points along the continuum, but Taxman and Bouffard [17] advocate for a "service delivery system that cuts across the different components of the criminal justice system" (p. 1684). Optimally, arrest would constitute the first point of contact for intervention.

In studies of offenders in treatment during various stages of the criminal justice continuum [18], or in various types of interventions [19], researchers have found that specific legal status was not as important a factor in treatment retention as was the individual's perceived level of threat. Furthermore, the level of perceived threat was not associated with legal status. Although there may be a presumption that the criminal justice system forces individuals to enter treatment, Wild and colleagues [10] found that 35% of legally mandated offenders perceived no coercion. Therefore, the presence and degree of perceived threat may not be related to a person's actual legal status, or where they are in the continuum [18,19]. In fact, some individuals may perceive arrest as threatening while others may not experience incarceration as threatening.

Using a community sample, Epstein and colleagues [10] examined the relationship between treatment admission and having an SUD. They found that of those with an SUD, those in treatment were three times more likely to be involved in the criminal justice system as compared with those who were not in treatment. They surmised, "when an adult becomes involved with the criminal justice system, factors related to coercion, adjudication, and alternative sentencing might override the effects or characteristics that were significant predictors of treatment receipt among the total population" (p. 864). Although this study compared CJ and non-CJ individuals on treatment entry, it selected only those with a current SUD rather than assessing the entire criminal justice sub population to determine who entered and who did not enter substance abuse treatment.

Similarly, national data sets report a high proportion of criminal justice referrals among those in treatment (i.e. Treatment Episode Data Set). Although these data sets allow for comparisons of the similarities and differences of CJ and non-CJ clients within treatment, they do not allow comparisons between those who entered treatment versus those that did not, nor do they allow for an assessment of the relationship between arrest and treatment admission.

One factor that may be influencing treatment entry is the severity of alcohol or other drug use. Severity of use has been associated with the perceived need for treatment in several studies of non-offending and offending populations [20-24]. However, in a study of 275 individuals referred for drug treatment, Hser and colleagues (1998) did not find severity of use a significant predictor of treatment entry, even when considering criminal justice status. They did find that family issues and an increasing number of psychological problems were negatively associated with treatment admission, while legal status – defined broadly as probation, parole, awaiting trial or sentencing – had a strong positive association with treatment admission [21]. Unfortunately, since the operational definition of legal status spanned several dispositions across the criminal justice continuum, we do not know the probability of treatment admission for the largest criminal justice group – those arrested – or how that probability varies by criminal justice dispositions further along the criminal justice continuum.

In general, predictors of admission into mental health and substance abuse treatment include age, sex, and marital status [20,25]. More specifically, women, those in the middle age ranges, and those who have been married were more likely to utilize services. In addition, those who received mental health services were more likely to receive substance abuse treatment [20] and those with a drug abuse disorder had a higher number of mental health visits than those with any other mental health disorders [25]. Although individuals in racial or ethnic minorities are less likely to receive mental health services [25], there are no differences between those who do and do not receive substance abuse treatment [20].

The goal of this exploratory study is to examine the relationship between arrest and admission to substance abuse treatment in a nationally representative, community-based sample. To do this we employ three models to examine characteristics of those entering specialty treatment; initially examining the general population, then comparing those arrested and finally examining difference among criminal justice dispositions. The first aim is to determine if there is an association between arrest and treatment and if it is independent of demographic factors. The second aim is to determine if the likelihood of treatment involvement varies for individuals who are in the community and involved in other criminal justice dispositions (i.e. probation and parole).

## Results

In 2002, nearly 5% of the adult sample (*n *= 1,684 of 36,370), collected as part of the National Survey on Drug Use and Health (NSDUH), was arrested and booked within the last 12 months. Because of the complex sampling design of the survey, we are able to extrapolate to the US population, projecting that nearly 6 million adults in the US were arrested in one 12-month period. Of those arrested, 41% were charged with either a drug or alcohol related offense. Those with at least one arrest were more likely to be male (OR = 3.74), to have a serious mental illness (SMI) (OR = 2.52), to be in a minority racial or ethnic group (OR = 1.49), to be younger (OR = 3.98) and to not be married (OR = 4.39). In addition they were more likely to have used an illegal substance in the past year (53.7% vs. 13.1%; OR = 7.68) and have an SUD (51.7% vs. 13.5%; OR = 10.33) than those who had not been arrested.

However, among those with a recent arrest, 38% (*n *= 643) were also under supervision (e.g. probation or parole). Similarly 2% (*n *= 757) of those in the general population and without a recent arrest were also under CJ supervision. To capture the nuances of the criminal justice continuum, two additional groups were formed (Arrest with Supervision and Supervision Only) and compared to the Non-CJ and Arrest Only groups (see Methods for details). There were highly significant differences between the four groups on all the demographic variables, serious mental illness (SMI), and prevalence of substance abuse or dependency (See Table 1).

**Table 1:** Comparison of characteristics of general population and four groups based on criminal justice disposition

	General Adult Population (*N *= 36,370)	Non-CJ (*n *= 33,929)	Arrest Only (*n *= 1041)	Arrest with CJ Supervision (*n *= 643)	CJ Supervision (*n *= 757)	Design-based *F *(*df*)	*p*-value*
Male	46.5	46.8	76.7	78.0	67.7	62.52	.001
Age							
18–25	14.7	13.8	38.8	40.7	30.1		
26+	85.3	86.2	61.2	59.3	69.9		
							
White	70.4	72.0	50.2	56.0	53.3	32.83	.001
Married	56.6	57.9	25.4	20.6	30.6	60.64	.001
Serious Mental Illness	11.0	8.0	19.0	16.4	14.0	23.46	.001
							
Any SUD	8.4	8.1	44.8	55.1	22.2	321.99	.001
Treatment Admission	1.3	0.5	9.1	24.0	10.7	450.45	.001

Note: Based on Design Based *F *Value; *df *= (1,900). Sample sizes are actual numbers; percentages weighted. CJ = criminal justice. For clarity the groups are: 1) Non-CJ – no arrest w/in last 12 months, no CJ supervision; 2) Arrest Only – arrest within last 12 months, no CJ supervision; 3) Arrest with Supervision – arrest within last 12 month with CJ supervision (probation/parole); 4) CJ supervision only – no recent arrest (last 12 months), but on probation or parole.

Because the prevalence of having an SUD varied from 8.1% (non-CJ group) to 55.1% (Arrest & Supervision group), we also compared a sub-sample of the four groups – those with an SUD – on the same variables (See Table 2). We continued to find significant differences on all of the variables except for the presence of an SMI. Among those with an SUD, there were no statistical differences on having symptoms of an SMI between those without criminal justice involvement or those with any of the three criminal justice dispositions.

**Table 2:** Comparison of demographics and treatment experience of those with a substance use disorder between each criminal justice group

	Non-CJ Population w/SUD (*n *= 4,488)	Arrest Only w/SUD (*n *= 511)	Arrest w/CJ supervision w/SUD (*n *= 359)	CJ supervision w/SUD (*n *= 210)	*F*	*p*-value
Male	64.2	82.1	75.5	79.2	11.83	.001
Age						
18–25	32.1	41.7	41.4	43.8	5.58	.001
26+	67.9	8.3	58.6	56.2		
White	71.7	58.4	63.2	52.7	6.52	.001
Currently Married	35.6	18.5	15.0	13.4	11.22	.001
Serious Mental Illness	19.7	25.6	25.0	21.8	1.47	.222
Treatment Admission	4.1	14.4	34.4	12.7	59.19	.001

Note: Based on Design Based *F *Value; *df *= (2.96, 2667.95). Sample sizes actual numbers; percentages weighted. CJ = criminal justice, SUD = substance use disorder.

To understand the association between treatment admission and arrest, we constructed three different logistic regression models that are consistent with the concept of the criminal justice system as a continuum. In the first model, we constructed a logistic regression model with demographic and individual characteristics as possible predictors to determine what factors were associated independently with any individual being admitted into specialty treatment (Table 3). In Model 1, having an SUD was most robustly associated with treatment (OR = 12.33 95% CI 8.89–17.10). Other significant associations included being male (OR = 1.39; 95% CI 1.01–1.93) and having an SMI (OR = 2.81, 95% CI: 1.97–4.02). Additionally, those currently married, as opposed to those unmarried (OR = 0.36; 95% CI 0.25–0.52) and those 18–25 years old, as compared with being older (OR = 0.49; 95% CI 0.36–0.65) were less likely to enter treatment.

**Table 3:** Logistic regression models predicting admission into specialty treatment.

	MODEL 1 OR (95% CI)	MODEL 2 OR (95% CI)	MODEL 3 OR (95% CI)
Sex			
Female (ref)	1.00	1.00	1.00
Male	1.39* (1.01–1.93)	1.10 (0.77–1.57)	0.98 (0.67–1.43)
Age			
18–25 (ref)	1.00	1.00	1.00
26-up	0.49***(0.36–0.66)	0.42***(0.31–0.58)	0.40***(0.29–0.55)
Race			
Non White (ref)	1.00	1.00	1.00
White	0.92 (0.68–1.26)	1.12 (0.81–1.56)	1.18 (0.84–1.64)
Marital Status			
Not Married (ref)	1.00	1.00	1.00
Married	0.36*** (0.25–0.52)	0.41***(0.28–0.62)	0.48***(0.32–0.73)
Serious mental illness			
No (ref)	1.00	1.00	1.00
Yes	2.81***(1.97–4.02)	2.70***(1.83–3.99)	2.74***(1.79–4.19)
SUD			
No (ref)	1.00	1.00	1.00
Yes	12.34***(8.90–17.10)	822***(5.79–11.67)	7.48***(5.14–10.87)
Arrest in last 12 mon.			
No (ref)	-------------------	1.00	-------------------
Yes		8.74***(5.80–13.18)	
CJ Status			
No CJ Status (ref)			1.00
Arrest Only	-----------------		7.19*** (3.94–13.10)
Supervise Only			15.26***(8.27–28.13)
Arrest & Supervise			22.62***(13.47–37.99)

Note: CJ = criminal justice, SUD = substance use disorder. 'Ref' refers to the reference category.

** *p *< .01, *** *p *< .001. All p-values are from Wald tests.

For Model 2, an arrest within the last 12 months was added to the model and age (OR = 0.42; 95% CI 0.31–0.58), marital status (OR = 0.41, 95% CI 0.28–0.62), and having an SMI (OR = 2.71, 95% CI 1.83–3.99) remained significant. Although the presence of an SUD remained significant, the magnitude of its association declined (OR = 8.22; 95% CI 5.79–11.67). Interestingly, having an arrest in the past 12 months increased the likelihood of receiving treatment similar in magnitude to that of having an SUD (OR = 8.74; 95% CI 5.80–13.18).

For Model 3, we constructed a variable that defined specific criminal justice status more thoroughly. This categorical variable depicts four groups with more distinct levels of criminal justice disposition (e.g. no involvement, arrest only, arrest with supervision and supervision only). The no criminal justice involvement group is the reference group. Age (OR = 0.40; 95% CI 0.29–0.55), marital status (OR = 0.48; 95% CI 0.32–0.73), SMI (OR = 2.74; 95% CI 1.79–4.19) and having an SUD (OR = 7.48; 95% CI 5.14–10.87) remained associated with treatment admission (See table 3). The association with the Arrest Only group (OR 7.18; 95% CI 2.94–13.10) remained significant compared to the referent group of no-CJ involvement. Those in the Supervision Only group occupied an intermediate position for entering treatment (OR = 15.26; 95% CI 8.27–28.12). The Arrest & Supervision group was the most likely to enter treatment than those in the non-CJ group (OR = 22.62, 95% CI 13.47–37.99).

### Additional descriptive analyses

We also examined the different types of treatment defined as 'specialty treatment.' We found that within each specific treatment location the proportion of participants attending were similar between the Arrest Only and non-CJ groups: Hospital based (42% of the non-arrest group and 39% of arrest group); Residential rehabilitation (48% of non-arrest and 48% arrest); Outpatient Rehabilitation (61% of non-arrest and 56% of arrest) and Mental Health Clinic (49% of non-arrest and 29% of arrest).

To further assess those who were involved in treatment without an SUD we restricted our analyses to the subpopulation of those who reported ***receiving specialty treatment ***in the past year (1.3%, *n *= 483 overall), irrespective of meeting criteria for a SUD. We found that 73.6% of those in the Supervision Only group entered specialty treatment without meeting diagnostic criteria for a current SUD compared to 36% of those in the non-CJ group, 29% of the Arrest Only and 21% of the Arrest & Supervision group.

Although funding for treatment may influence who attends versus who does not, it appears that the Arrest Only group may utilize different types of funding when compared to the no-CJ group. Those with arrest only were more likely to use public assistance (35.6% versus 19.9%) and military benefits (19.7% versus 12.0%) for treatment funding compared to those not involved in criminal justice. Conversely the non-CJ group was more likely to have their treatment funded by Medicaid (23.3% versus 10.6%) and employment related benefits (13.3% versus 5.6%) than the Arrest Only group. Certainly funding available through the courts may also be a catalyst for treatment admission among those with an arrest than those without. We found that court funding for treatment was involved in 22.1% of those in the Arrest Only group. Surprisingly, 6.5% of those claiming not to have a recent arrest or under CJ supervision also claimed treatment funding from the courts. Other sources of court funding may include Probate, Family or Mental Health Courts.

## Discussion

This study examined the association between arrest and admission to specialty drug treatment using a community dwelling nationally representative sample. As such, the study initially focused on the independent contribution of arrest to the probability of a treatment admission in the same year. Finally, the analyses examined if the association between arrest and treatment admission was similar to or different from the association with treatment admission at other points in the criminal justice continuum.

Self reported arrest in the NSDUH appears valid. According to the Criminal Justice Sourcebook [26], there were 8.2 million adult arrests reported in 2002. Our projections based on the NSDUH data indicate that nearly 6 million individuals accumulated almost 7.8 million arrests in this same year. The remaining differences could be attributed to sampling error, method for collection of arrest data in the NSDUH (maximum of three arrests tallied), under-reporting of arrests by individuals, or that some of the arrested population is likely imprisoned and not part of the survey population. Demographic characteristics between criminal justice data and those reporting criminal justice involvement in the NSDUH sample were also similar, particularly the predominance of white males.

Our findings supports previous findings of the high proportion of drug and alcohol involvement among those involved in the criminal justice system [4,20,22]. The arrest group was much more likely to have used an illegal substance in the past year, meet criteria for drug or alcohol dependency and enter specialty treatment than the non-arrest group. This trend remained consistent even when restricting analysis to only those meeting diagnostic criteria for SUD.

However, the examination of arrest versus non-arrest may obscure differences in legal status that might influence treatment entry, conflating what might be individual factors with system factors on treatment admission. Replacement of the two-level arrest/non-arrest variable with the four-level categorical variable representing current criminal justice status in the analyses did not change the individual factors predicting treatment (i.e. unmarried, SMI, and SUD). In fact, examination of multiple groups underscores that those involved in the criminal justice system, irrespective of the point in the continuum, are more likely to be admitted to treatment than those who are not. However, the variation in the likelihood of treatment involvement among those points in the continuum is cause for further examination.

The odds of being admitted into treatment for those in the Arrest Only group are seven fold that of those in the non-CJ group, even though they may not be processed through the continuum further. Although we cannot precisely determine the temporal ordering of arrest and treatment, it is possible that the perceived threat associated with arrest may motivate individuals to enter treatment independent of the coercion associated with mandated treatment through formal sentencing. However perceived threat may increase as legal status changes since those in the Arrest Only group were the least likely among the CJ groups to be admitted into specialty treatment even though the proportion of those who met criteria for an SUD was similar to Arrest & Supervision (45% and 55% respectively) and greater than Supervision Only (22%).

One explanation for those in the Arrest & Supervision group being most likely to receive treatment is the greater level of scrutiny they experience through their involvement in the criminal justice system. Hypothetically as they move from arrest to conviction and sentencing, they experienced court ordered assessments and investigation and multiple court appearances. The recent experience of criminal justice processing may achieve the highest level of perceived threat among the dispositions studied and thus induce the highest level of motivation to participate in treatment.

In a recent study of drug-using offenders on probation, Longshore & Teruya [27], like others [20] found a significant relationship between motivation and external pressure in the form of criminal justice coercion. However, Longshore and Teruya [27] argue that treatment motivation is better understood as two related but distinct constructs – readiness and resistance. Readiness, as the positive side of motivation, was a significant predictor of treatment retention during the 6-month period after intake. Resistance, or opposition to treatment, is considered the negative side of motivation and predicted drug use. The authors point out that opposition to treatment can be based upon sceptical views of treatment or in the case of legal mandate – a resentment over the loss of individual control over decision making.

Considering the dual constructs within measurement of motivation forces us to assess not only how and when coercion is used, but also contemplating how to enhance treatment readiness among offending populations. If treatment is unwarranted or the offender becomes oppositional to treatment – even though they enter – we may have squandered important resources. For example, those in the Supervision Only group were most likely to be admitted to specialty treatment without a diagnosis of either abuse or dependency. It could be that a history of a drug or alcohol problem combined with a recent relapse was the catalyst for a mandated or voluntary treatment admission. Alternatively, treatment could be a proxy for a criminal justice sanction, with treatment involvement as an inducement for avoiding confinement. Conversely, confinement may obscure time frames associated with diagnosing a current SUD. Those in jail or prison for an extended period of time would not report recent use, and hence not meet DSM diagnostic criteria for a substance use disorder simply because they did not have the opportunity to use. However, further studies are needed to examine if treatment entrance was clinically appropriate or more associated with legal coercion.

Inappropriate admission into specialty treatment is not isolated to the intersection between criminal justice and treatment. Our findings suggest, similar to others [25], that those in the general population are admitted to treatment without meeting diagnostic criteria or when they have relatively mild manifestations of their disorder. In fact, among the four groups, the non-CJ group was second highest among those entering treatment without a SUD diagnosis. This finding may reflect difficulties in relying on self-report, determining clinical diagnosis from structured interviews, or people obtaining treatment inappropriate to the level of care required [28]. However, limiting our analysis to only those who met current diagnostic criteria may have obscured this finding.

Although we speculated that informal social controls such as family relationships might be predictive of treatment admission, marriage did not positively influence treatment admission in any of our models. Reflecting other studies [29], we found those who were married were less likely to enter treatment than those who were not. Certainly a dichotomous variable does little to inform us as to the quality of the relationship or the influence an individual may have in coercing their spouse into treatment. However, in another study, a more qualitative measurement of relationship that captured some of these additional marital elements (i.e. support) yielded similar results [20]. Furthermore, others have found that marriage may be protective of continued drug use and criminal activity, particularly among offending populations [30].

Finally, it should be noted that individuals with an SMI were more likely to be in criminal justice populations than in the general population and they comprised approximately 20% of those with a SUD in each of the offending and non-offending groups. The co-occurrence of SMIs and SUDs has been well documented [31-33], but it is of particular concern when the individual is involved in the criminal justice system due to the inattention of co-occurring disorders in both the substance abuse and mental health treatment systems [32,33]. A supervising agent making a referral to a mental health provider may assume that substance abuse issues would be addressed, or vice versa. Treatment non-compliance and/or a misunderstanding of the interaction of the two disorders may result in a higher level of confinement (jail or prison) than appropriate treatment would indicate. Although it is noteworthy that SMI predicts receiving specialty treatment across models, these multiple morbidities (SMI, SUD, and CJ) require highly specialized services that integrate mental health and substance abuse treatment while simultaneously integrating the requirements and potential constraints that the criminal justice system imposes [34].

### Limitations

The self-report nature of the data may underestimate socially sanctioned behaviors such as arrest and drug use resulting in lower bound estimates. In addition, the NSDUH survey samples individuals living in the community, and therefore we are missing the subpopulation of those who are arrested and incarcerated. Although a small percentage of those arrested actually go to state and federal prisons, it potentially excludes those who have committed more serious offenses and who may have more serious and chronic substance abuse problems [35].

Another limitation is our inability to know the temporal sequences. We do not know if the arrest or treatment came first; we only know that both treatment and arrest occurred within the same year. Unfortunately, other potentially useful datasets such as the Treatment Episode Data Set and the now defunct Arrestee Drug Abuse Monitoring program either do not include "arrest" as a referral mechanism or do not have community comparison groups [4,36].

In addition, we do not know the sequence of events for people who have both arrest and criminal supervision within the same year. Certainly our assumption that they have been convicted of the offense they were arrested for and are now serving out their sentences in the community under probation/parole supervision is probable: there is no data, however, to confirm it. Consequently the four group categorizations were derived to focus on those who clearly identify as those at the arrest phase of the criminal justice continuum without including those under supervision. Finally, this study did not control for treatment access and other barriers to treatment which may have influenced the results if known.

## Conclusion

Although involvement across all points in the criminal justice system is associated with a higher probability of admission into specialty treatment, only 16% of those recently arrested with an SUD received treatment in the same year as their arrest. Our findings support others that suggest that only a fraction of those needing treatment in the CJ system receive it [37-39]. Conversely, we found that in the CJ and non-CJ groups many were admitted into specialty substance abuse treatment that did not meet diagnostic criteria for any SUD. While these findings may be similar to other general population surveys [25], the judicious use of treatment resources for criminal justice populations seems especially prudent. Because the need is so great, and the opportunity for intervention at hand, more precise use of screening, assessment and placement criteria [28] is essential to efficiently use available resources.

The focal point for this study was arrest. Although arrest involves the largest number of individuals entering the criminal justice system, it is also the most fleeting point as individuals can move in and out rather quickly. Minimally, arrest imposes contact between the individual and a law enforcement person and can be an opportunity for early intervention strategies such as pre-arraignment diversion into treatment [12] or brief intervention strategies. Brief interventions, defined as one-time contacts that can be limited to 5 minutes, have been found effective in reducing use or moving individuals forward in the intervention process in a number of settings [40]. Because brief interventions have been used effectively in various non-traditional settings, it may be effective in this setting [41]. Moreover, using brief intervention at this early point in the criminal justice continuum may motivate a greater number of individuals to seek treatment or decrease use and perhaps prevent further involvement with the criminal justice system.

We also found that assessing arrest versus non-arrest, rather than considering the criminal justice continuum, obscures differences in treatment involvement at various points in the criminal justice system. Although the criminal justice system may both motivate treatment readiness and create opposition to treatment through coercion [27] researchers and practitioners need to treat individuals at various points in the continuum as heterogeneous. Legal status is dynamic, changing with circumstances, time and offender behavior. As legal status shifts, the motivation for treatment can also shift from an individual's desire to put their life back together after an arrest to external coercion via a legal mandate to participate in treatment while in prison or on probation. Capitalizing on the possibility of motivation post arrest, by using arrest as a catalyst for enhancing treatment readiness, could prove to increase secondary prevention efforts and keep more individuals out of confinement. Certainly this strategy is more fiscally and resource prudent. Multiple arrests, court processing and incarceration costs are far greater than those associated with treatment. Likewise enhancing treatment readiness – rather than contributing to oppositional behavior within treatment – can produce more favourable treatment outcomes decreasing not only use and abuse of substances, but criminal behavior.

## Methods

For this secondary analysis, we used the 2002 National Survey on Drug Use and Health (NSDUH) – formerly the National Household Survey on Drug Abuse). The NSDUH is a comprehensive national survey that provides quarterly as well as annual estimates of self reported drug use and treatment involvement and is conducted yearly by the Substance Abuse and Mental Health Services Administration (SAMHSA). It measures the prevalence and correlates of drug use, as well as, treatment history and questions on mental disorders that allow for diagnostic criteria to be applied. In addition, respondents are asked about personal and family income, health care access, illegal activities and problems associated with the use of drugs. The multistage area probability sampling frame, for each of the 50 states, includes the civilian, non-institutionalized population aged 12 and older. In 2002, there was a screening response rate of 79% yielding 54,079 records in the public use file. Because of the differences between juvenile and adult criminal justice systems, the analysis is limited to the subpopulation of adults over the age of 18 (unweighted sample size is 36,370). (See ICPSR website at the University of Michigan for further information.)

Individuals were counted as having been arrested if they had a response of one or more to the following question: "Not counting minor traffic violations, how many times during the past 12 months have you been arrested and booked for breaking a law." Current criminal justice supervision was determined through two questions: 1) Were you on probation at any time during the past 12 months? 2) Were you on parole, supervised release, or any other conditional release from prison at any time during the past 12 months? An affirmative answer to either of these question was coded as 'currently under criminal justice supervision.' As those arrested may also have been on parole or probation, we then divided the sample into 4 groups: 1) *Non-CJ *– those with no criminal jusitce involvement in the last 12 months; 2) *Arrest Only *– those with an arrest in the last 12 months; 3) *Arrest & Supervision *– those with a recent arrest and on probation or parole; and, 4) *Supervision Only *– those with no recent arrest but under criminal justice supervision.

Measures for this analysis include demographics (age, race, gender, marital status), current arrest and criminal justice supervision status during the past 12 months; substance abuse and or dependency diagnosis; serious mental illness (SMI) and admission to a speciality substance abuse treatment program during the past 12 months. Diagnostic criteria from the Diagnostic and Statistical Manual (DSM) of Mental Disorders IV [42] is imbedded within the survey allowing for classification of the substance use disorders (SUD) of abuse or dependence. SMI is determined using the K-6 [43]. The six items related to symptoms of psychological distress in the previous 12 months, are scored on a 5-point Likert scale from 0 (none of the time) to 4 (all of the time). The range of summed scores is 0 – 24 with scores of 13 or above classified as indicating an SMI [44].

Measurement of treatment involvement was restricted to admission into 'speciality' treatment in the past 12 months. Speciality treatment is defined as treatment delivered by a professional in one of four settings: outpatient clinic, residential facility, mental health facility or acute care hospital.

Descriptive and inferential statistics are used to compare the two groups of arrest and non-arrest and then the four groups differing on criminal justice status and supervision level. Additional descriptive analyses, conducted in an effort to understand additional factors that may be associated with treatment admission, examined treatment admission without an SUD, the type of specialty treatment or treatment location and funding for treatment.

To determine factors independently associated with treatment admission we used binomial and multinomial logistic regression models. Three specific regression models are used to understand the association between treatment admission and various criminal justice positions (including no involvement). This is consistent with the concept of the criminal justice system as a continuum. Levels of court interaction and/or supervision vary throughout the continuum potentially varying the level of perceived coercion or threat. The three models are as follows: Model 1) Determination of factors associated independently with any individual being admitted into specialty substance abuse treatment; Model 2) Determination of how the incidence of an arrest within the past 12 months is associated with treatment independent of other factors; and Model 3) Determination of how arrest compares with other criminal justice dispositions in its association with treatment admission.

All analyses adjusted for the complex survey design by including sampling weights, primary sampling units, and strata in the statistical models using the survey procedures associated with the Taylor Series Expansion [45] available in StataSE 9 (StataCorp, College Station, TX). Unweighted counts are included in the tables, but sample weights were used for all analyses. The magnitudes of association are reported as odds ratios (OR) with 95% confidence intervals (95% CI).

## Competing interests

The author(s) declare that they have no competing interests.

## Authors' contributions

SPK drafted and revised the manuscript, participated in the study design, statistical analysis, and interpretation of results.

CLA participated in the study design, statistical analysis, and interpretation of results and revising the manuscript.

JAS was responsible for conceptualization of the four groups, provided technical assistance on the criminal justice system and assisted in revising.

ALK participated in the literature review and revising the manuscript.
